# Exploring Blastocystis: hidden complexity in a common intestinal microbe

**DOI:** 10.1099/jmm.0.002198

**Published:** 2026-10-07

**Authors:** Isabel Guadano-Procesi, Alma García Ramos, Eleni Gentekaki, Anastasios D. Tsaousis

**Affiliations:** 1 Department of Clinical Sciences and Translational Medicine, Tor Vergata University of Rome, 00133 Rome, Italy; 2 Instituto Universitario de Enfermedades Tropicales y Salud Pública de Canarias (IUETSPC), Universidad de La Laguna, 38029 La Laguna, Tenerife, Spain; 3 Department of Veterinary Medicine, University of Nicosia, Nicosia 2414, Cyprus; 4 Laboratory of Molecular and Evolutionary Parasitology, School of Natural Sciences, University of Kent, Canterbury CT2 7NJ, Kent, UK

**Keywords:** gastrointestinal manifestation, gut microbiome, protist, subtypes

## Abstract

*Blastocystis* is an anaerobic stramenopile that colonizes the large intestine of humans and many animals worldwide. Small-subunit rRNA-based typing reveals deep genetic diversity: at least 44 genetic variants, namely, subtypes (STs), are recognized, with ST1–ST4 accounting for most human carriage. Its medical relevance remains contested. *Blastocystis* carriage is frequently asymptomatic, and while associations with non-specific gastrointestinal symptoms (e.g. diarrhoea, abdominal pain and bloating), irritable bowel syndrome and extraintestinal manifestations such as urticaria have been proposed. In parallel, multiple microbiome studies, including a recent population-scale analysis, link *Blastocystis* carriage to higher bacterial diversity, healthier diets and favourable cardiometabolic profiles, suggesting that in many settings, it may be a marker of a resilient gut ecosystem rather than a primary pathogen. Transmission is primarily faecal–oral, most likely via environmentally resilient cysts, with potential for zoonotic and waterborne spread. Diagnosis using quantitative PCR is the most sensitive method; microscopy has limited sensitivity and can be confounded by morphological plasticity, though its diagnostic accuracy varies considerably according to the experience of the microscopist. Treatment is typically based on excluding alternative causes rather than confirmed causality; while metronidazole is commonly used, clinical outcomes are highly variable due to potential co-infections, inconsistent links between parasite eradication and symptom relief and reports of reduced drug susceptibility.

## Graphical Abstract

Graphical overview of the present *Blastocystis* review: historical perspective, clinical presentation, microbial characteristics, putative pathogenic strategies, epidemiology, diagnosis and biosafety, treatment and resistance and key open questions.

## Historical perspective

*Blastocystis* was first described in the early 20th century. Despite this, its taxonomic position was debated for decades. *Blastocystis* was variously interpreted as a cystic protozoan [1, 2], a yeast-like organism [3] and even a fungus [4], largely because of its non-specific and poorly distinctive appearance in stool microscopy. Phylogenetic analysis ultimately placed *Blastocystis* within the stramenopiles (a large and diverse eukaryotic supergroup), and current taxonomy recognizes the genus *Blastocystis* accordingly (class Bigyra; order Opalinata; family Blastocystidae) [5]. Species designations based on host (e.g. *Blastocystis hominis*) are no longer recommended for mammalian and avian isolates; instead, isolates are referred to as *Blastocystis* sp. and classified into subtypes based on small subunit (SSU) rRNA sequence divergence [6].

## Clinical presentations

*Blastocystis* carriage is common and frequently incidental. When symptoms are attributed to *Blastocystis*, they are typically non-specific and overlap with those seen in many gastrointestinal conditions, such as diarrhoea, abdominal discomfort, bloating, flatulence, nausea and altered bowel habit [7]. Associations have been reported with irritable bowel syndrome (IBS) and, less consistently, inflammatory bowel disease (IBD), as well as extraintestinal manifestations, most often urticaria and pruritus (for review, see [7]). To date, these associations remain largely observational, and causality has not been established. Interpretation is complicated due to (i) frequent co-detection of other enteric pathogens, (ii) host factors (e.g. immune status, pre-existing gut inflammation and diet) and (iii) parasite diversity. In practice, detection should be interpreted in clinical context rather than treated as proof of causation [8].

## Microbial characteristics

### Phenotypic features

*Blastocystis* is pleomorphic. Vacuolar and cyst forms are most commonly described; granular and amoeboid forms are reported more variably, especially in culture [9]. The cyst form is widely considered the most likely transmissible stage because of its environmental resilience. *Blastocystis* is non-motile and lacks flagella [10]. It is adapted to low-oxygen conditions and harbours mitochondrion-related organelles [11] with a reduced respiratory chain, including an alternative oxidase that may buffer transient oxygen exposure [12].

### Genotypic features

*Blastocystis* genomes are compact (up to 20 Mb; ~6,000 predicted genes) yet highly divergent between subtypes [13, 14]. At least 44 subtypes have been recognized to date [15], and ST1–ST4 comprise the majority of human isolates in most surveys [16]. The depth of divergence among subtypes supports the view that they may represent separate species, a key consideration for diagnostics, epidemiology and treatment response. Consequently, exploring potential intra-subtype biological variation alongside standard subtype classifications may further complement our understanding. Expanding future research towards strain-level (Fig. 1) resolution could help refine clinical and ecological insights.

**Fig. 1.**
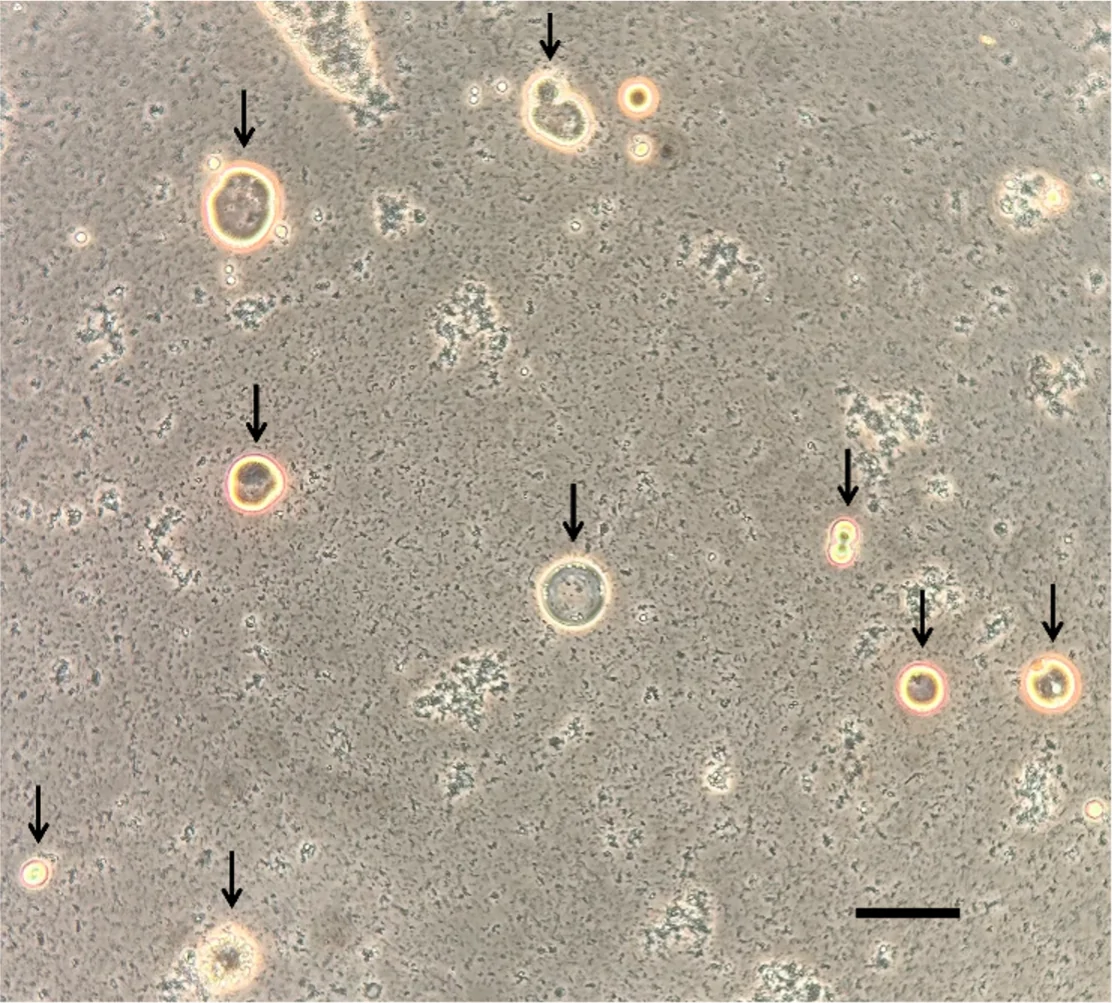
Bright-field light micrograph of *Blastocystis* subtype ST3 maintained under xenic culture conditions. The isolate was molecularly identified as ST3 by PCR amplification and sequencing of the SSU rRNA gene before cultivation. Arrows indicate representative vacuolar forms of *Blastocystis*. The presence of associated bacterial flora is consistent with xenic cultivation and reflects the typical growth environment employed for the propagation of ST3 isolates. The vacuolar and granular forms shown are commonly observed across *Blastocystis* subtypes and are not subtype-specific. Image acquired using a 40× objective (scale bar: 20 µm).

## Laboratory confirmation and safety

### Specimen type

The primary specimens for the detection of *Blastocystis* are fresh (Fig. 1) stool samples, as it predominantly inhabits the gastrointestinal tract. For research purposes, isolates can also be obtained through *in vitro* cultures of stool to allow further phenotypic or genotypic analyses [17]. In epidemiological studies, samples may additionally include faecal material from animal hosts or environmental sources, depending on the scope of the investigation. Specimen collection should ensure minimal contamination and preserve viability when culturing is intended.

### Laboratory confirmation

A combination of microscopic, culture-based and molecular approaches can be used:

- Microscopy: wet mounts and permanent stains can detect characteristic forms, but sensitivity is modest and interpretation is microscopist dependent.
- Culture: xenic culture can enrich *Blastocystis* and increase sensitivity, enabling downstream phenotypic work; however, culture conditions can bias subtype recovery.

- Molecular methods: PCR targeting the SSU rRNA gene (with sequencing) provides the most reliable confirmation and allows subtype assignment; while quantitative PCR is increasingly used in epidemiological and clinical settings due to its high detection sensitivity and throughput, sequencing-based approaches are still required for definitive subtype-level characterization.

### Laboratory safety

Handling *Blastocystis* specimens requires biosafety level 2 (BSL-2) precautions, in line with international guidelines. The manipulation of stool samples and cultures of *Blastocystis* should follow Centers for Disease Control and Prevention (CDC) recommendations for BSL-2 practices, which include the use of gloves, lab coats and eye/face protection when there is a risk of splashes or aerosols [18]. The UK Health Security Agency (UKHSA) guidance recommends that any laboratory procedure likely to generate infectious aerosols be performed inside a microbiological safety cabinet [19]. The World Health Organization (WHO) emphasizes adherence to standard precautions for pathogens of faecal origin. These include proper handling of specimens, surface disinfection, autoclaving of waste and strict compliance with personal protective equipment protocols [20]. Overall, strict adherence to these biosafety measures ensures the protection of laboratory personnel while allowing reliable confirmation and characterization of *Blastocystis*.

## Treatment and resistance

### Treatment

There is no universally accepted treatment algorithm because causality is often uncertain and most carriage is asymptomatic. When treatment is pursued (typically for persistent symptoms after exclusion of other causes), metronidazole remains the most commonly prescribed drug, sometimes in combination with agents such as paromomycin or trimethoprim–sulfamethoxazole [21]. Nitazoxanide, tinidazole and iodoquinol have also been used, with variable success across studies. It should be noted, however, that much of the current therapeutic literature relies on relatively small or observational datasets. The current scarcity of large-scale, randomized controlled trials introduces some limitations when defining standardized treatment strategies. Consequently, practical clinical guidance emphasizes that detection should not trigger automatic eradication; clinicians should instead prioritize evaluating concurrent disorders (like IBS or IBD) and strictly screen faecal microbiota transplantation (FMT) donors before pursuing targeted therapy.

### Resistance

Reduced *in vitro* susceptibility to metronidazole has been reported, and clinical responses are inconsistent across studies [22, 23]. Differences in drug response between subtypes (and potentially within-subtype strain variation) may contribute to treatment failure. Improved links between subtype/strain typing and clinical outcomes are needed before susceptibility-guided management becomes realistic [21]. Recent studies have also demonstrated that *Blastocystis* might have a potential mechanism of resistance to both chlorine and hydrogen peroxide [24].

## Pathogenic strategies

### Host range

*Blastocystis* colonizes a broad range of hosts (including livestock, companion animals and wildlife), positioning it at a critical intersection of human, animal and environmental health (One Health). Subtype distribution varies by host species and setting, creating potential for zoonotic exchange and environmental persistence [25, 26]. Recent molecular evidence of its presence in soil further highlights an environmental transmission route, with shared human and environmental subtypes adding complexity to its epidemiology [27, 28]. Notably, host-adapted physiological differences and variable colonization dynamics across hosts challenge basic generalizations regarding its transmission cycle. Consequently, identical subtype findings alone cannot definitively establish direct zoonotic directionality, reinforcing the need for a truly integrated One Health approach [29].

### Vectors and sylvatic cycle

This parasite is not transmitted by vectors; rather, it exhibits a broad host range, including birds, primates, rodents, reptiles, amphibians, fish and insects. The maintenance of a sylvatic cycle relies heavily on these diverse wildlife reservoirs, creating persistent environmental pools that bridge natural ecosystems with domestic and agricultural settings.

### Virulence factors

*In silico* predictions identified multiple cysteine proteases and hydrolytic enzymes, some of which were tested *in vitro* and in laboratory animal experiments. While these experimental models report effects such as degradation of secretory IgA 30[30, 31], disruption of epithelial tight junctions, increased intestinal permeability, induction of apoptosis and modulation of inflammatory signalling, none of these mechanisms have yet been demonstrated in humans [30]. This highlights a substantial *in vitro–in vivo* evidence gap, as experimental models often lack the complex physiological barriers of the human gut. Evidence suggests that these effects may be subtype- and strain-dependent and may also depend on the resident bacterial community [32].

## Epidemiology

### Transmission

*Blastocystis* is transmitted via the faecal–oral route. While cyst and vacuolar forms of this parasite can be shed, the cyst is widely recognized as the principal infectious and environmentally resilient stage responsible for transmission [33]. Transmission can occur through multiple routes, direct or indirect. Direct transmission includes human-to-human, human-to-animal or animal-to-human contact, while indirect transmission occurs through the ingestion of water or food contaminated with faecal matter [28].

### Infection

After ingestion, *Blastocystis* colonizes the large intestine and may persist for extended periods as carriage. The infectious dose and incubation period remain poorly defined, and environmentally resilient cysts are presumed to be the principal infectious stage. Because carriage is common and co-detection with other enteric pathogens is frequent, *Blastocystis* detection in stool should be interpreted alongside symptoms, alternative diagnoses and host context (e.g. gut inflammation, immune status and diet).

### Epidemiology

*Blastocystis* is one of the most common parasites colonizing the gastrointestinal tract of humans and a wide range of non-human animals, with a cosmopolitan distribution worldwide [34]. In fact, this protist is estimated to be present in over one billion individuals. Reported prevalence varies substantially across regions, often exceeding 50% in developing countries and detected in both symptomatic and asymptomatic individuals [35]. A global metagenomic study of 56,989 individuals across 32 countries reported that *Blastocystis* carriage varies with geography and lifestyle and is positively associated with healthier dietary patterns, with prevalence also increasing in a diet-improvement intervention [36]. While these findings highlight a strong link to gut health on a population level, ongoing research aims to clarify how these favourable associations might be modulated by specific subtype variations or host-specific contexts and the potential confounding effects of diet or lifestyle in these large data cohorts remain to be fully clarified.

### Risk groups

Most people carrying *Blastocystis* are asymptomatic. Where symptoms occur, they are more often reported in contexts that increase exposure or vulnerability, although causality remains uncertain and co-infections are common. These include the following:

- People living in or travelling to settings with limited sanitation or unsafe drinking water, including humanitarian and crowded living settings.
- Young children (including daycare settings) where faecal–oral transmission is more likely.
- Individuals with frequent contact with animals or animal faeces (livestock, companion animals, wildlife interfaces, slaughterhouses) and those exposed to manure-contaminated environments.
- People with underlying gastrointestinal disorders (e.g. IBS/IBD) or immunocompromising conditions may be more likely to be investigated and treated, but the clinical significance of detection is variable.

### Prevention

Prevention is primarily based on standard water, sanitation and hygiene and food safety measures. Because carriage is common and often incidental, preventive advice should focus on exposure reduction rather than eradication.

- Use safe drinking water (treated municipal supply, bottled or boiled/filtered water where safety is uncertain).
- Promote effective sanitation and safe disposal of human waste; avoid sewage or septage contamination of water sources.
- Hand hygiene after toilet use, changing nappies, animal handling and before preparing food.
- Food hygiene: wash and, where appropriate, peel produce; avoid consuming raw foods washed with untreated water.
- Animal-associated exposures: use protective measures when handling manure and clean potentially contaminated surfaces; manage manure runoff from farms.
- In clinical practice, interpret *Blastocystis* detection in the context of symptoms, alternative diagnoses and co-infections; approaches to FMT donor screening vary and should follow local guidance.

## Open questions

- Which *Blastocystis* subtypes/strains (if any) are consistently linked to defined clinical syndromes, and what host factors modify this relationship?
- What is the full *in vivo* life cycle, including the infectious dose and the relative contribution of cyst versus other forms to transmission?
- How do *Blastocystis* and bacterial community structure influence one another, and when is carriage a marker of gut ‘eubiosis’ versus a contributor to symptoms?
- Which molecular mechanisms underpin epithelial barrier effects and immune modulation observed *in vitro* and do these occur *in vivo*?
- How should clinicians interpret *Blastocystis* detection in IBS/IBD workups and in screening of FMT donors?
- Can subtype/strain-resolved diagnostics and standardized culture tools improve reproducibility across laboratories and enable susceptibility testing?

## Abbreviations

BSL-2: biosafety level 2
CDC: Centers for Disease Control and Prevention
FMT: faecal microbiota transplantation
IBD: inflammatory bowel disease
IBS: irritable bowel syndrome
SSU rRNA: small subunit rRNA
ST: subtype
UKHSA: UK Health Security Agency
WHO: World Health Organization
